# The SON_2_A_2_ score: A novel grading scale for predicting hemorrhage and outcomes after thrombolysis

**DOI:** 10.3389/fneur.2022.952843

**Published:** 2022-10-31

**Authors:** Yu Ren, Zhongxiang He, Xiaoyan Du, Jie Liu, Li Zhou, Xue Bai, Yue Chen, Bowen Wu, Xiaosong Song, Libo Zhao, Qin Yang

**Affiliations:** ^1^Department of Neurology, Nanchong Central Hospital, Sichuan, China; ^2^Health Manage Center, The Second Affiliated Hospital of Chongqing Medical University, Chongqing, China; ^3^Department of Neurology, The Yongchuan Hospital of Chongqing Medical University, Chongqing, China; ^4^Chongqing Key Laboratory of Cerebrovascular Disease Research, Chongqing, China; ^5^Department of Neurology, The First Affiliated Hospital of Chongqing Medical University, Chongqing, China; ^6^Department of Neurology, The Ninth People's Hospital of Chongqing, Chongqing, China

**Keywords:** ischemic stroke, symptomatic intracranial hemorrhage, thrombolytic therapy, risk score, retrospective cohort study

## Abstract

**Objectives:**

This study aimed to develop a score including novel putative predictors for predicting the risk of sICH and outcomes after thrombolytic therapy with intravenous (IV) recombinant tissue-type plasminogen activator (r-tPA) in acute ischemic stroke patients.

**Methods:**

All patients with acute ischemic stroke treated with IV r-tPA at three university-based hospitals in Chongqing, China, from 2014 to 2019 were retrospectively studied. Potential risk factors associated with sICH (NINDS criteria) were determined with multivariate logistic regression, and we developed our score according to the magnitude of logistic regression coefficients. The score was validated in another independent cohort. Area under the receiver operating characteristic curve (AUC-ROC) was used to assess the performance of the score. Calibration was evaluated using the Hosmer–Lemeshow goodness-of-fit method.

**Results:**

The SON_2_A_2_ score (0 to 8 points) consisted of history of **s**moking (no = 1, yes = 0, β = 0.81), **o**nset-to-needle time (≥3.5 = 1,<3.5=0, β = 0.74), **N**IH Stroke Scale on admission (>10 = 2, ≤10 = 0, β = 1.22), **n**eutrophil percentage (≥80.0% = 1, <80% = 0, β = 0.81), **A**SPECT score (≤11 = 2, >11 = 0, β = 1.30), and **a**ge (>65 years = 1, ≤65 years = 0, β = 0.89). The SON_2_A_2_ score was strongly associated with sICH (OR 1.98; 95%CI 1.675–2.34) and poor outcomes (OR 1.89; 95%CI 1.68–2.13). AUC-ROC in the derivation cohort was 0.82 (95%CI 0.77–0.86). Similar results were obtained in the validation cohort. The Hosmer–Lemeshow test revealed that predicted and observed event rates in derivation and validation cohorts were very close.

**Conclusion:**

The SON_2_A_2_ score is a simple, efficient, quick, and easy-to-perform scale for predicting the risk of sICH and outcome after intravenous r-tPA thrombolysis within 4.5 h in patients with ischemic stroke, and risk assessment using this test has the potential for early and personalized management of this disease in high-risk patients.

## Introduction

From 2013 to 2019, the prevalence of stroke in China increased significantly from 2.28–2.58% (1), posing a serious challenge to the public health, and a broad-based nationwide strategy in stroke prevention, screening, and consulting as well as effective intervention is urgently needed. Intravenous thrombolysis within 4.5 h of symptom onset with recombinant tissue-type plasminogen activator (r-tPA) has been proven to be the most effective evidence-based medical treatment for acute ischemic stroke patients (2). Nevertheless, not all individuals benefit from the thrombolytic therapy, due to narrow therapeutic windows and severe treatment complications. What clinicians and patients' dependents fear most of r-tPA treatment is thrombolysis-related symptomatic intracranial hemorrhage (sICH). The reported frequencies of sICH differ between trials according to the definition selected (3, 4). Post-thrombolytic sICH, a life-threatening intracerebral hemorrhage, alters the outcomes of acute ischemic stroke patients, resulting in a high in-hospital mortality and disability at discharge (5). Therefore, stratification of the risk of sICH might facilitate patient selection for thrombolytic therapy.

Increasing evidence showed that baseline factors and individual variables played a predominant role in affecting the risk of post-thrombolytic sICH (5–11). These include baseline National Institutes of Health Stroke Scale (NIHSS) score (6–11), ethnicity (6), gender (6, 9), age (6–11), high blood pressure (6, 10), high baseline serum glucose (6–8, 10, 11), and onset-to-treatment time (OTT) (10, 11), all of which are immediately available at emergency department. Based on the above factors, several scales including GRASPS scores (6), HAT (7), SEDAN (8), THRIVE (9), SITS-SICH (10), DRAGON (11), and other particular scores, in which each variable was ascribed according to its weight in the nomogram (12), had been proposed for predicting the risk of sICH following thrombolysis. The predictive performance of these risk scores has been externally validated and compared, and they appear to have fairly good predictive power (13). However, none of them has been extensively used in clinical practice for some reasons.

Alberta Stroke Program Early CT score is a generally accepted predictor for both functional outcomes and symptomatic hemorrhage in AIS patients, which has never been incorporated into score systems. It was found that in patients with an extended time window, the incidence of sICH was similar among NCCT ASPECT score, CTP, and MRI-guided endovascular treatment population (14). It may indicate that ASPECT score is associated with ischemic penumbra and collateral status (15). Moreover, ASPECT score can be immediately obtained by prethrombolysis noncontrast CT, which is less time-consuming and cost-effective. Therefore, we included ASPECT score as a putative predictor into the score system.

In this study, we aimed to develop and validate a simple and reliable scoring tool for predicting the risk of sICH and outcomes in AIS patients with IVT in Chinese population, which may help certain patients receiving IVT avoid fatal thrombolytic complications. We present the SON_2_A_2_ score, derived from our multi-center cohorts for acute AIS patients treated with IV r-tPA.

## Methods

### Derivation cohort

**Figure 1:**
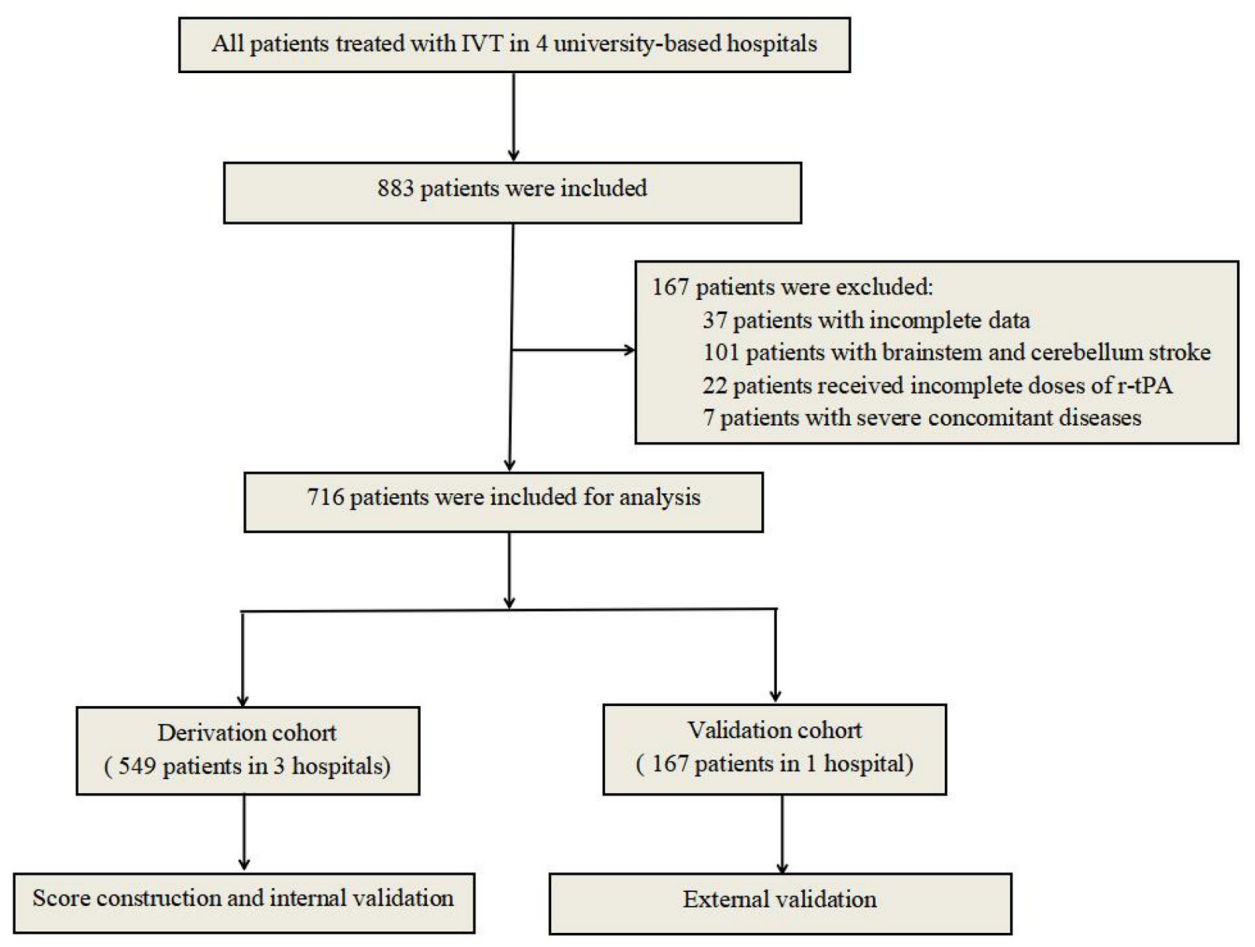
Flowchart of our study.

All patients with acute ischemic stroke treated with IV r-tPA from June 2014 to June 2019 at the First Affiliated Hospital of Chongqing Medical University, Chongqing, China, the Second Affiliated Hospital of Chongqing Medical University, Chongqing, China, and the Yongchuan Hospital of Chongqing Medical University, Chongqing, China, were included in this study. The inclusion criteria were as follows: (1) All patients had to meet the diagnostic criteria of acute ischemic stroke according to Guidelines in China 2018; (2) R-tPA was administrated at the standard dose (0.9 mg/kg of body weight) within 4.5 h after the onset of symptoms; (3) cerebral CT scans were performed at admission and within 24 h after thrombolysis or whenever ICH was suspected; and (4) all patients had to be hospitalized more than 3 days [almost all sICHs occurred within 36 h after thrombolysis (16)] or diagnosed as sICH. Patients with bridging therapy (IVT followed by endovascular treatment) were not included in our population. The exclusion criteria were as follows: (1) patients with incomplete data; (2) patients with brainstem and cerebellum stroke (the accuracy of the ASPECT score may be influenced by the structure of basalis skull); (3) patients receiving incomplete pre-calculated doses of r-tPA; and (4) patients with severe concomitant diseases, such as severe heart, liver, or kidney diseases or systemic diseases. A research flowchart is demonstrated in Figure 1.

### Construction of scoring system

Baseline demographics, vascular risk factors, medical history, stroke type, baseline NIHSS score, OTT, laboratory parameters, and radiologic data of the eligible patients were obtained by reviewing electronic medical records. The ASPECT score based on noncontrast CT before treatment was reviewed by two independent neuroradiologists without any knowledge of patients, and inter-rater reliability was tested by Spearman's rank correlation coefficient; any dispute was resolved by negotiation. Functional outcome at discharge was evaluated using mRS. Good short-term outcome was defined as mRS score 0 to 2, and poor short-term outcome was defined as mRS score 3 to 6. Eligible patients were dichotomized into two groups, namely, the non-sICH and sICH group, according to the NINDS criteria. Independent risk factors were considered to be predictors of sICH, and point values assigned to predictors were based on their magnitude of regression coefficients by rounding off to the nearest integer value. For each patient, the total risk score was calculated as the sum of points assigned to the predictors.

### Internal cross-validation with bootstrap and external validation

After the SON_2_A_2_ score system was established, internal cross-validation of the regression model between parameters of the SON_2_A_2_ score and short-term outcome was performed based on 1,000 bootstrap replicates. External validation was performed in an independent cohort of 160 patients receiving thrombolysis from the Ninth People's Hospital of Chongqing, Chongqing, China (2014–2019). All the patients in the validation cohort met the same inclusion and exclusion criteria of the derivation cohort.

### Statistics

We performed descriptive statistics for all available baseline variables including patients with or without sICH. Normally distributed continuous variables were presented as the mean ± SD, and continuous variables with abnormal distribution were presented as the median (IQR). Categorical variables were presented as percentages. Differences between sICH and non-sICH groups were compared using Student's *t*-test or Mann–Whitney U test for continuous variables and Pearson's χ^2^ tests or the Fisher's exact test for categorical variables, as appropriate. Continuous data were divided into two categories using receiver operating characteristic curve (ROC) combined with clinical practicality. Univariate logistic regression was used to identify risk factors for sICH, and variables associated with sICH at the *P* ≤ 0.10 level in the univariate analysis were incorporated as potential predictive factors into the multivariate logistic regression model. In this analysis, independent risk factors for sICH were determined by a backward regression procedure. Confounding factors were excluded in the backward regression procedure. This process is presented in Supplementary Table.

We stratified the total risk scores into the following four tiers according to the predicted probability: low, moderate, high, and extremely high risk. Then, binary logistic regression was conducted to test the efficiency of the SON_2_A_2_ score in predicting short-term outcomes. The discrimination capacity of the risk score was assessed by area under the receiver operating characteristic curve (AUC-ROC), and calibration was evaluated using the Hosmer–Lemeshow goodness-of-fit method. Statistically significant differences were set at *P* < 0.05. All analyses were performed using SPSS statistical software version 24.0 for Windows.

## Results

A total of 883 acute ischemic stroke patients treated with IV r-tPA met the inclusion criteria, and 167 patients were eventually excluded for not meeting the predetermined study criteria. Finally, 716 patients (395 males, 55.2%) were eligible for analysis. Prevalence of sICH was 10.3% (95%CI 8.3–12.3%) according to the NINDS criteria (91 of 883). The overall median age was 72 years (IQR, 63–78 years), the median baseline NIHSS score was 11 (IQR, 5-17), and the median time from symptom onset to therapy was 2.5 h (IQR, 2.0–3.2 h). The median mRS at discharge was 2 (IQR, 1-4). The inter-rater reliability was 0.82, indicating high inter-rater consistency and reliability. The detailed baseline characteristics of the patients in the derivation and validation cohorts are presented in Tables 1, 2, respectively.

**Table 1:** Demographics and baseline characteristics of patients with and without sICH in the derivation cohort.

**Characteristics**	**Non-sICH**	**sICH**	***P***
	***n* = 488**	***n* = 68**	
Age, median, IQR (years)	71 (63, 77)	74 (69, 80)	0.001
Sex, Male,%	287, 58.8%	26, 38.2%	0.010
OTT, hour, median, IQR (hours)	2.5 (2, 3)	2.9 (2, 3.5)	0.035
NISHSS, median, IQR	10 (5, 16)	17 (14, 20)	0.000
Hypertension,%	254, 52.0%	44, 64.7%	0.050
Diabetes,%	96, 19.7%	13, 19.1%	0.914
Atrial fibrillation,%	118, 24.2%	28, 41.2%	0.030
Stroke history,%	90, 18.4%	8, 11.8%	0.176
Statin,%	86, 17.6%	11, 16.2%	0.768
Antithrombotic drugs,%	103, 21.1%	18, 31.0%	0.315
smoking,%	183, 37.5%	11, 16.2%	0.010
Drinking,%	147, 30.1%	13, 19.1%	0.060
Leukocyte, median, IQR (10^9^/L)	7.66 (6.31, 8.9)	7.85 (6.23, 9.76)	0.457
Neutrophil percentage, M ± SD	68.83 ± 12.50	72.63 ± 13.06	0.009
Hb, median, IQR(g/L)	136 (126, 145)	133 (124, 141)	0.039
PLT, median, IQR(10^9^/L)	188 (150, 222)	174 (135, 198)	0.014
PT, median, IQR(s)	12.7 (11.7, 13.5)	13.1 (12.4, 14.1)	0.005
APTT, median, IQR(s)	30.3 (26.1, 34.5)	32 (28.4, 35.0)	0.060
INR, median, IQR	1.02 (0.96, 1.10)	1.05 (0.98, 1.12)	0.116
Fibrinogen, median, IQR(g/L)	2.94 (2.50, 3.42)	2.80 (2.26, 3.38)	0.199
ASPECTS, median, IQR	13 (12, 14)	12 (10, 13)	0.000
mRS, median, IQR	2 (0, 4)	5 (4, 6)	0.000

SD, Standard Deviation; IQR, Interquartile Range; ASPECTS, Alberta Stroke Program Early CT score; PT, Prothrombin Time; APTT, Activated Partial Thromboplastin Time; INR, International Normalized Ratio; mRS, modified Rankin Scale.

**Table 2:** Demographics and baseline characteristics of patients with and without sICH in the validation cohort.

**Characteristics**	**Non-sICH**	**sICH**	***P***
	***n* = 146**	***n* = 14**	
Age, median, IQR (years)	72 (65, 79)	76 (66, 79)	0.921
Sex, Male, %	77, 52.7%	5, 35.8%	0.233
OTT, hour, median, M ± SD (hours)	2.91 ± 0.98	3.31 ± 1.35	0.161
NISHSS, median, IQR	10 (6, 16)	19 (15, 20)	0.000
Hypertension, %	90, 61.6%	11, 78.6%	0.210
Diabetes, %	28, 19.2%	3, 21.4%	0.839
Atrial fibrillation, %	29, 19.9%	3, 21.4%	0.889
Stroke history, %	20, 13.7%	6, 42.9%	0.005
Statin, %	11, 7.5%	3, 21.4%	0.079
Antithrombotic drugs, %	13, 8.9%	4, 28.6%	0.315
smoking, %	55, 37.7%	5, 35.7%	0.940
Drinking, %	41, 28.1%	3, 21.4%	0.594
Leukocyte, median, IQR (10^9^/L)	6.79 (5.64, 8.17)	6.92 (5.15, 8.81)	0.738
Neutrophil percentage, M ± SD	69.89 ± 11.12	68.65 ± 21.96	0.837
Hb, median, IQR (g/L)	134 (126, 145)	133 (124, 141)	0.331
PLT, median, IQR (10^9^/L)	178 (147, 228)	143 (100, 175)	0.003
PT, median, M ± SD (s)	11.3 ± 0.8	11.5 ± 0.8	0.394
APTT, median, IQR (s)	25.7 (23.9, 28.0)	26.6 (24.6, 29.0)	0.221
INR, median, IQR	0.98 (0.92, 1.09)	1.04 (0.93, 1.18)	0.481
Fibrinogen, median, IQR (g/L)	2.74 (2.40, 3.05)	2.53 (2.26, 3.03)	0.197
ASPECTS, median, IQR	13 (13, 14)	11 (10, 12)	0.000
mRS, median, IQR	0 (1, 4)	5 (4, 5)	0.000

SD, Standard Deviation; IQR, Interquartile Range; ASPECTS, Alberta Stroke Program Early CT score; PT, Prothrombin Time; APTT, Activated Partial Thromboplastin Time; INR, International Normalized Ratio; mRS, modified Rankin Scale.

Compared with the counterparts, patients with sICH in the derivation cohort tended to be older (74 vs. 71, *P* < 0.01), were more likely to have a longer time delay from stroke attacks (2.9 vs. 2.5, *P* < 0.05) and a higher NIHSS score (17 vs. 10, *P* < 0.01), as well as were inclined to have a poorer ASPECT score (12 vs. 13, *P* < 0.01). Additionally, patients with an elevated neutrophil percentage, prolonged PT, and reduced blood platelet count were more frequent to progressing to sICH (72.63% vs. 68.83%, *P* < 0.01, 13.1 vs. 12.7, *P* < 0.01, and 174 vs. 188, *P* < 0.05, respectively). The risk of sICH according to gender, medical comorbidities, and medication use is illustrated in Table 1. In univariate logistic regression, sex, age, hypertension, atrial fibrillation, smoking history, drinking history, OTT, NIHSS, ASPECTS, neutrophil percentage, PT, and blood platelet count showed an association with sICH. After adjusting for confounding variables, age, smoking history, baseline NIHSS, OTT, neutrophil percentage, and ASPECTS independently predicted sICH in multiple logistic regression. Regression coefficients and point value assigned to predictors are illustrated in Table 3.

**Table 3:** SON_2_A_2_ score (0–8) for predicting the risk of symptomatic intracranial hemorrhage after IV r-tPA and the final regression model.

**Predictors**	**Category**	**Points**	**Regression coefficients**	***P***
**Smoking**	Yes	0	Reference	-
	No	1	0.81	0.03
**O**nset–to–needle time (hours)	<3.5	0	Reference	–
	≧3.5	1	0.74	0.03
**N**IHSS score	≦11	0	Reference	–
	>11	2	1.22	<0.01
**N**eutrophil percentage	<80%	0	Reference	–
	≧80%	1	0.81	<0.01
**A**ge (years)	≦65	0	Reference	–
	>65	1	0.89	0.03
**A**SPECT score	>11	0	Reference	–
	≦11	2	1.30	< 0.01

**Figure 2:**
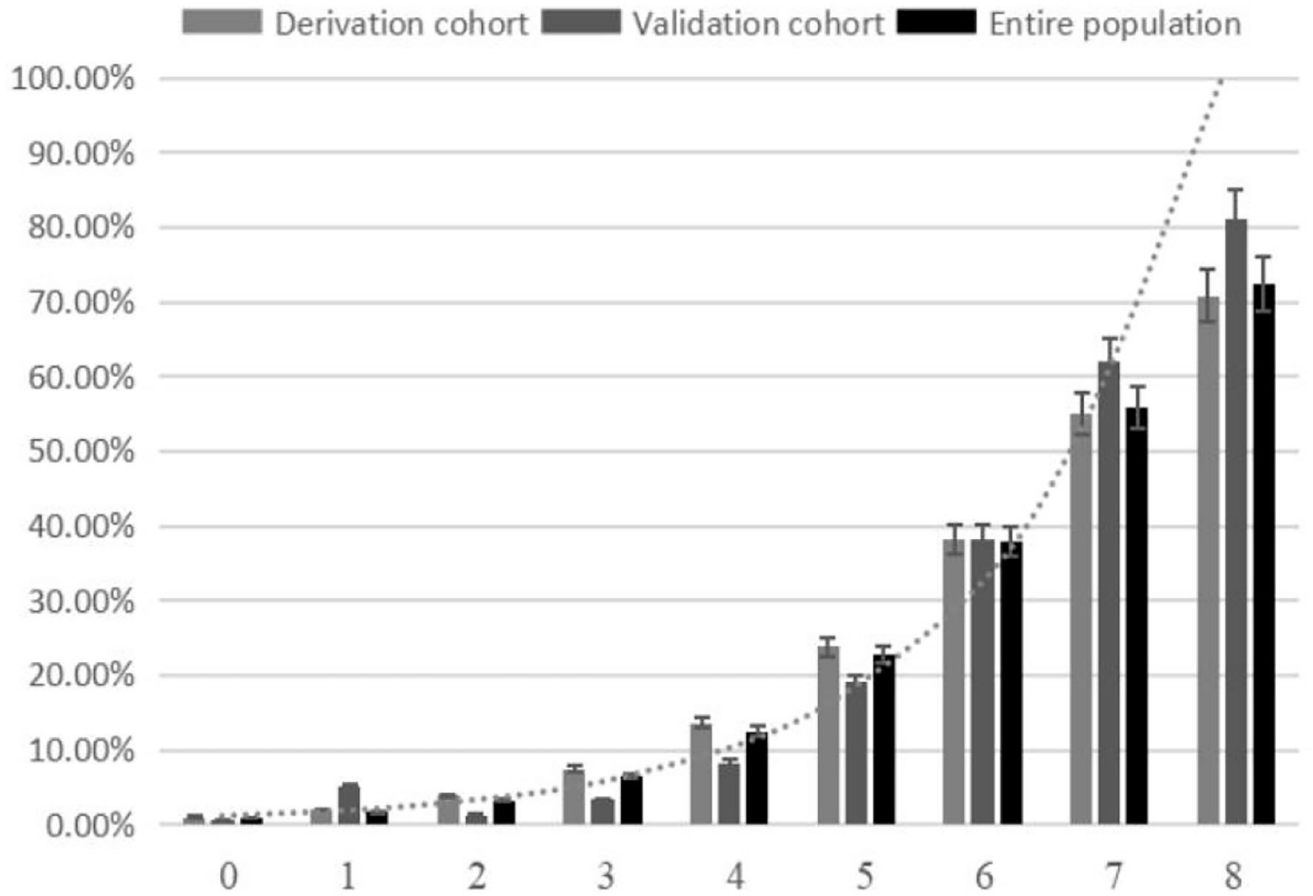
Risk of sICH per SON_2_A_2_ score point.

**Figure 3:**
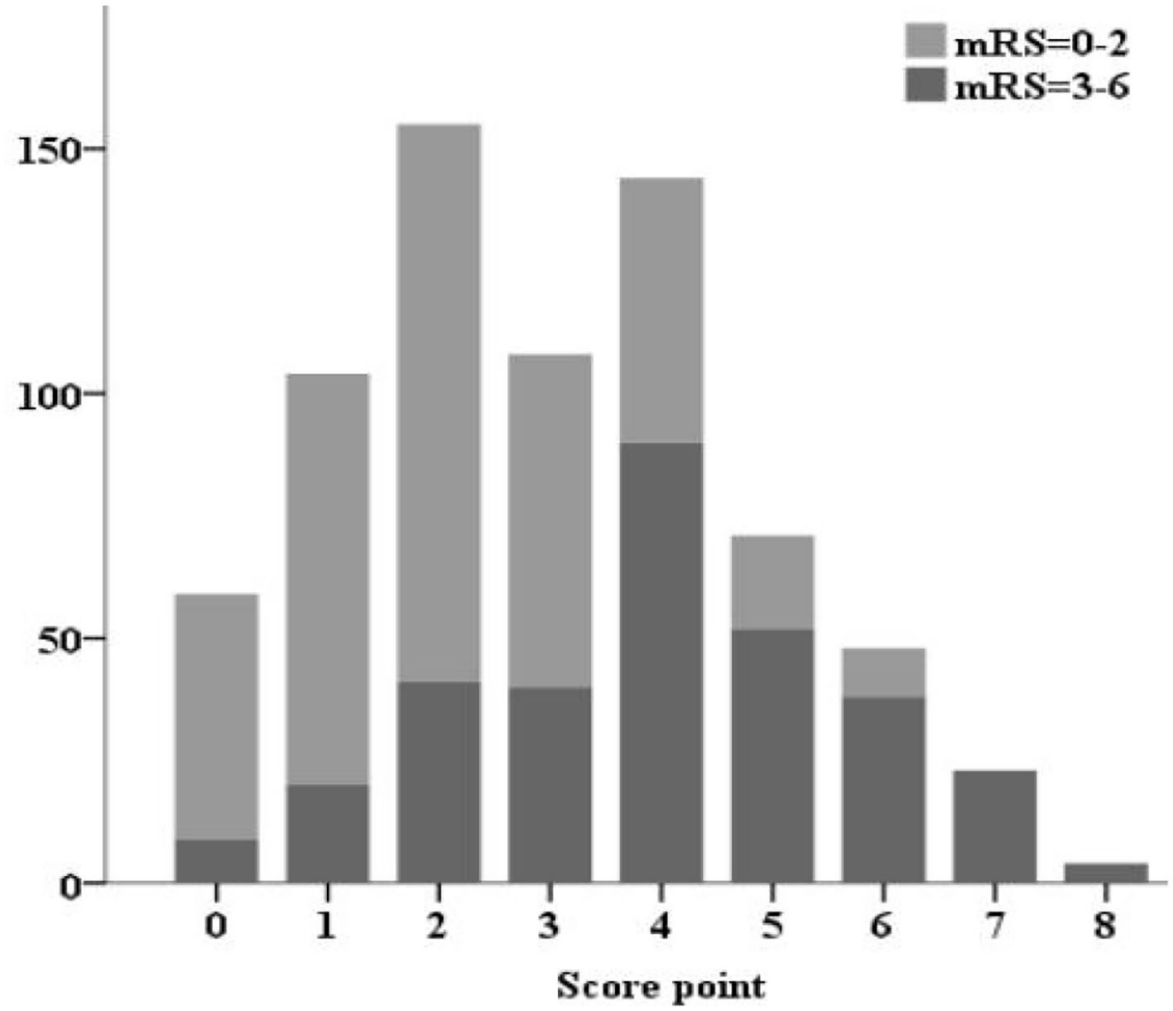
Proportion of patients with good and poor outcomes for each SON2A2 point.

**Figure 4:**
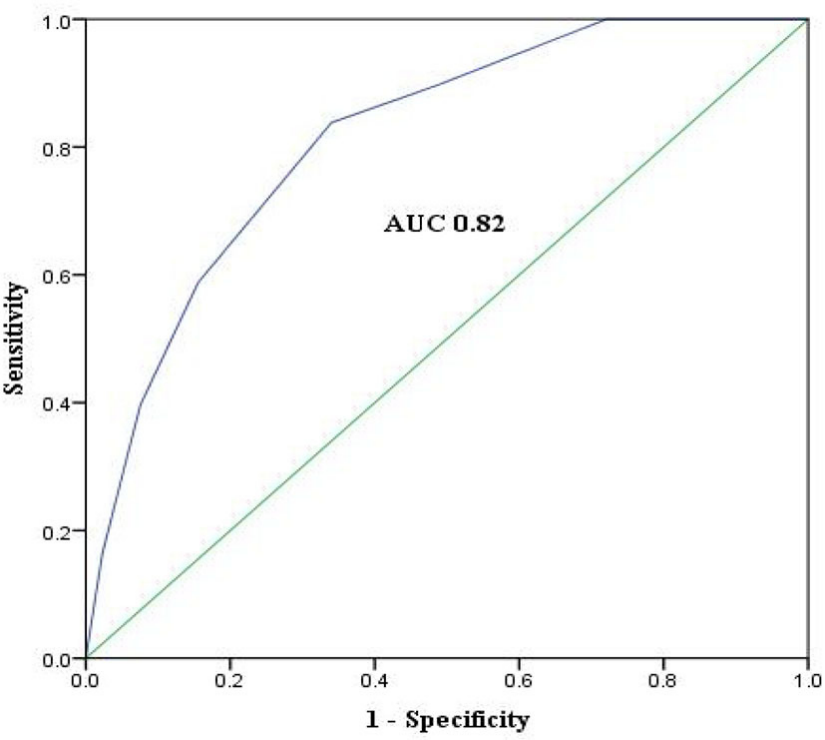
ROC curves of the derivation cohort.

The SON_2_A_2_ score, calculated as the sum of each predictor's scores, ranged from 0 to 8. A strong association between SON_2_A_2_ score and sICH was shown in a binary logistic regression procedure in the derivation, validation, and the entire population (OR 1.98; 95% CI, 1.67–2.34, *P* < 0.01; OR 2.63; 95% CI, 1.67–4.13, *P* < 0.01; OR 2.07; 95% CI, 1.77–2.43, *P* < 0.01, respectively). Prediction probability for sICH per increasing point in the above three cohorts is shown in Figure 2. The median SON_2_A_2_ score was 3, and the best threshold for the SON_2_A_2_ score to diagnose sICH was 3.5 with a positive likelihood ratio of 2.46. Moreover, our risk scores strongly correlated with poor short-term outcomes in the entire population (OR 1.89; 95% CI 1.62–2.10, *P* < 0.01). The proportion of patients with good and poor outcomes for each SON_2_A_2_ point is illustrated in Figures 3, 4. Based on the prediction probability, the SON_2_A_2_ score was divided into four levels, which are 0–1, 2–4, 5–6, and 7–8 for low (1.7%), moderate (7.4%), high (26.9%), and extremely high risk (62.7%), respectively.

Discrimination power of the score evaluated by AUC-ROC was similar in the derivation cohort, the internal cross-validation cohort, and the external validation cohort (AUC 0.82, 95%CI 0.77–0.86, *P* < 0.01, vs. 0.82, 95%CI 0.77–0.87, *P* < 0.01, and 0.88, 95%CI 0.80-0.96, *P* < 0.01). ROC curve of the derivation cohort is presented in Figure 4. The Hosmer–Lemeshow test revealed that predicted and observed event rates in the derivation and validation cohorts were very close (χ^2^ = 4.6, df = 5, *P* = 0.47 vs. χ^2^ = 0.61, df = 5, *P* = 0.99), indicating that the model was well calibrated.

## Discussion

In this study, we identified independent risk factors for sICH and on this basis we developed and validated a risk score for predicting sICH and outcomes after thrombolysis. The SON_2_A_2_ score comprised history of smoking, ASPECTS, onset-to-therapy time, baseline NIHSS score, neutrophil percentage, and age. All of these factors are part of routine assessments for r-tPA treatment candidates, which can be easily and rapidly determined at emergency departments. And this study was the first one to include ASPECTS as a predictor to develop a risk score. For bedside practicality, we also converted continuous variables into categorical variables and obtained cutoff values for each variable, making the score easy-to-perform. The AUC-ROC of 0.82 indicates the score has good discriminatory capacity to predict sICH. Our score also has good calibration, which implies the predicted incidence of sICH is consistent with that of the observed incidence. Moreover, the SON_2_A_2_ score strongly correlated with discharge mRS, signifying it has good predictability of short-term functional outcomes. Similar results of external validation support the generalization of the score. If confirmed in prospective studies, it is expected to be widely used in clinical practice.

Apart from an elevated percentage of neutrophils and history of smoking, the remaining components of the SON_2_A_2_ score had been reported to be independent risk factors for sICH (5–11). In a previous study, Gautier and his coworkers found pharmacological depletion of polymorphonuclear neutrophils reduced the risk of ICH, in parallel with a decrease in endothelial dysfunction in cerebral blood vessels (17). Moreover, Maestrini et al. reported higher neutrophil counts independently related to sICH and worse outcome (18). Unfortunately, no similar results were obtained in our study. However, a weak correlation was found between neutrophil percentage and sICH in univariate analysis (crude OR 1.03; 95%CI 1.00–1.05, *P* < 0.05). We subsequently dichotomized neutrophil percentage into more than and equal to 80% and <80%, and then, we found a high neutrophil percentage independently predicted sICH after adjusting confounders (adjusted OR 2.25; 95% CI 1.24–4.09, *P* < 0.05). Smoking is a recognized risk factor for ischemic stroke. To be intriguing, in our study history of smoking was found to be a protective factor for sICH. Coincidentally, smoking was independently associated with recanalization and reperfusion, indicating that thrombolytic therapy acts more effectively in smokers (19). This may be explained by smoking associated with increased plasma levels of carbon monoxide and episodic hypoxia, which could lead to ischemic preconditioning and may trigger adaptive cellular responses to ischemia (20).

High blood pressure and high blood glucose at admission before treatment had been confirmed to be associated with sICH and included in several risk scores as predictors (6–10). As a matter of fact, these studies only focused on the initial value at admission without longitudinal evaluation of blood pressure and blood glucose levels. As is well known, blood glucose and blood pressure may fluctuate dramatically over time and are probably merely a stress reaction after stroke (21, 22). For the absence of standard guidelines, clinicians may manage blood glucose and blood pressure at different levels at their discretion, which may modify the effect of blood pressure and hyperglycemia on outcomes after thrombolysis. It may prestroke glycemic variability and early-stage blood pressure variability, be associated with hemorrhagic transformation and worse outcomes (23, 24), and not necessarily be glucose and blood pressure at admission, in patients receiving intravenous thrombolysis. Hence, we think it is controversial to incorporate these two factors to develop a risk score, and more relevant studies are needed to clarify this issue. This also reminds us that blood glucose, blood pressure levels, and other indicators can be continuously monitored in the following research to obtain optimal cutoffs.

Stroke outcomes have improved in the past decade, caused by the improvements in in-hospital stroke care. According to a big data study from Singapore (25), there has been a decreasing incidence of AIS in Asia, but the rate of thrombolysis in Asian patients is still much lower than that in developed countries (26, 27) (9.5% in China vs. 11.7–18.2% in the USA). Due to poor or low r-tPA reperfusion rate and because patients receiving thrombolytic therapy have a higher ICH rate and consequently worse outcomes compared with the counterparts, it is necessary to identify patients who are more likely to develop sICH after thrombolysis. The SON_2_A_2_ score is strongly associated with sICH and poor outcomes; hence, we suppose our score system could facilitate patients selection. We classified the risk of sICH after thrombolysis into four levels, namely, low with SON_2_A_2_ score 0–1, moderate with SON_2_A_2_ score 2–4, high with SON_2_A_2_ score 5–6, and extremely high with SON_2_A_2_ score 7–8. The rate of sICH increased 37-fold and 8.5-fold, respectively, in patients with extremely high risk (62.9%), compared with those with low risk (1.7%) and moderate risk (7.4%). Therefore, we think it might not be rational to perform thrombolysis therapy among patients with extremely high risk of sICH. As for whether to withhold thrombolysis in patients with high risk (26.9%) of sICH, an assessment of potential net benefit to the patients is required.

According to the established score systems, for developing a clinical risk score for predicting the risk of post-thrombolytic sICH, any combinations of the following aspects of predictors could be used: (1) demographic characteristics; (2) medical history; (3) baseline neurological examination; (4) laboratory findings; (5) neuroradiologic features; and (6) specific therapy. To our knowledge, the more aspects a score covers, the higher accuracy and precision it may have. Compared with three aspects in GRASPS (6) and HAT (7) scores and four aspects in SEDAN (8) and SITS-SICH risk scores (10), our SON_2_A_2_ score comprises five aspects. This may be one of the possible reasons why our risk score has a higher AUC-ROC over other four scores (0.82 vs. 0.71, 0.72, 0.77, and 0.70, respectively). Without practice application and head-to-head comparison, we cannot say our scores have a better performance than other established scores. We have to declare that we do not propose withholding r-tPA treatment for patients at high risk of sICH according to the SON_2_A_2_ score before prospective evidence is available. However, clinicians could quantify risks based on our score and tell patients and their relatives what potential risks may involve in thrombolytic treatment. For patients at high risk of hemorrhagic transformation, more positive and effective medical care measures, such as longer stay in stroke unit, more frequent assessments of neurological deficit, and shorter CT scan intervals, should be taken.

This study has limitations attributed to its retrospective nature. All of our data came from teaching hospitals, and the number of patients experiencing ICH, especially sICH, was relatively small. We only included patients for whom all required elements were available, and 18.9% patients were excluded for not meeting the predetermined study criteria. These can only be addressed in a prospective study. Stratification of continuous variables and conversion of correlation coefficients to score point values, although convenient to clinical applications, are likely to cause a loss of information and decrease model accuracy.

## Conclusion

In conclusion, the SON_2_A_2_ score is easy to perform and time-saving, is well calibrated and validated, and has good predictive ability for the risk of sICH and outcomes in patients with ischemic stroke treated with IVT, providing clinicians, patients, and relatives an understanding of the risks involved in the current treatment.

## Data availability statement

The raw data supporting the conclusions of this article will be made available by the authors, without undue reservation.

## Author contributions

QY, XS, LZha, ZH, XD, and YR participated in the conception and design of the study. JL, LZho, XB, YC, and BW performed the data collection and analysis. YR wrote the first draft of the manuscript. All authors contributed to the study conception and design, commented on previous versions of the manuscript, and read and approved the final manuscript.

## Funding

This work was supported by grants from the National Natural Science Foundation of China (Grant nos. 82171456 and 81971229) and the Project of Science and Technology Strategic Cooperation between City and University (22SXQT0046).

## Conflict of interest

The authors declare that the research was conducted in the absence of any commercial or financial relationships that could be construed as a potential conflict of interest.

## Publisher's note

All claims expressed in this article are solely those of the authors and do not necessarily represent those of their affiliated organizations, or those of the publisher, the editors and the reviewers. Any product that may be evaluated in this article, or claim that may be made by its manufacturer, is not guaranteed or endorsed by the publisher.

## Abbreviations

AIS: acute ischemic stroke
AUC-ROC: area under the receiver operating characteristic curve
ASPECTS: Alberta Stroke Program Early CT score
mRS: modified Rankin Scale
NIHSS: NIH Stroke Scale
OTT: onset-to-treatment time
r-tPA: recombinant issue-type plasminogen activator
sICH: symptomatic intracranial hemorrhage.
