# Supplementary material for: The SON2A2 score: A novel grading scale for predicting hemorrhage and outcomes after thrombolysis
Source: Front Neurol. 2022 Oct 31;13:952843. doi: 10.3389/fneur.2022.952843 (PMC9659729; doi:10.3389/fneur.2022.952843)
Supplement: Supplementary file 1 [file Data_Sheet_1.PDF]

***Supplementary table*. Risk Factors in the Multivariate Logistic Regression.**

	**Risk factors**	**Regression coefficients**	**Odds Ratio**	**95% confidence interval**	***p* value**
Step 1	ASPECTS	1.212	3.362	1.83-6.17	0.000
	Neutrophil percentage	0.831	2.296	1.25-4.22	0.007
	OTT	0.679	1.973	1.00-3.88	0.049
	Age	0.823	2.277	0.98-5.29	0.056
	NIHSS	1.131	3.097	1.57-6.11	0.001
	Male	-0.286	0.751	0.35-1.62	0.464
	Hypertension	0.375	1.455	0.80-2.67	0.224
	Atrial fibrillation	0.401	1.494	0.82-2.73	0.191
	Drinking	-0.4	0.67	0.23-1.92	0.456
	PLT	-0.001	0.999	0.99-1.00	0.656
	PT	0.019	1.019	0.94-1.11	0.65
	Smoking	-0.876	0.416	0.14-1.22	0.111
Step 2	ASPECTS	1.223	3.397	1.85-6.22	0.000
	Neutrophil percentage	0.827	2.287	1.25-4.20	0.008
	OTT	0.699	2.013	1.03-3.93	0.041
	Age	0.842	2.321	1.00-5.37	0.049
	NIHSS	1.14	3.126	1.59-6.16	0.001
	Male	-0.264	0.768	0.36-1.64	0.495
	Hypertension	0.376	1.457	0.80-2.67	0.222
	Atrial fibrillation	0.42	1.522	0.84-2.76	0.167
	Drinking	-0.427	0.652	0.23-1.86	0.423
	PT	0.019	1.019	0.94-1.11	0.654
	Smoking	-0.918	0.399	0.14-1.15	0.09
Step 3	ASPECTS	1.221	3.392	1.85-6.21	0.000
	Neutrophil percentage	0.84	2.316	1.27-4.24	0.006
	OTT	0.71	2.035	1.04-3.97	0.037
	Age	0.853	2.347	1.02-5.42	0.046
	NIHSS	1.139	3.123	1.58-6.16	0.001
	Male	-0.262	0.77	0.36-1.64	0.499
	Hypertension	0.368	1.445	0.79-2.64	0.231
	Atrial fibrillation	0.428	1.535	0.85-2.78	0.158
	Drinking	-0.434	0.648	0.23-1.84	0.416
	Smoking	-0.91	0.402	0.14-1.16	0.092
Step 4	ASPECTS	1.209	3.349	1.83-6.13	0.000
	Neutrophil	0.844	2.326	1.27-4.25	0.006

	percentage				
	OTT	0.712	2.038	1.04-3.98	0.037
	Age	0.852	2.344	1.02-5.41	0.046
	NIHSS	1.175	3.239	1.66-6.34	0.001
	Hypertension	0.361	1.435	0.79-2.62	0.239
	Atrial fibrillation	0.419	1.521	0.84-2.76	0.167
	Drinking	-0.31	0.733	0.28-1.92	0.529
	Smoking	-1.009	0.365	0.13-1.00	0.049
Step 5	ASPECTS	1.238	3.45	1.90-6.27	0.000
	Neutrophil percentage	0.82	2.271	1.25-4.13	0.007
	OTT	0.7	2.015	1.03-3.93	0.04
	Age	0.862	2.367	1.03-5.46	0.043
	NIHSS	1.17	3.221	1.65-6.30	0.001
	Hypertension	0.341	1.407	0.77-2.56	0.262
	Atrial fibrillation	0.416	1.516	0.84-2.75	0.169
	Smoking	-0.798	0.45	0.21-0.95	0.037
Step 6	ASPECTS	1.295	3.652	2.03-6.58	0.000
	Neutrophil percentage	0.825	2.282	1.26-4.14	0.007
	OTT	0.741	2.097	1.08-4.07	0.028
	Age	0.906	2.475	1.08-5.69	0.033
	NIHSS	1.145	3.141	1.61-6.13	0.001
	Atrial fibrillation	0.366	1.442	0.80-2.59	0.221
	Smoking	-0.799	0.45	0.21-0.95	0.036
Step 7	ASPECTS	1.296	3.653	2.03-6.57	0.000
	Neutrophil percentage	0.813	2.254	1.24-4.09	0.007
	OTT	0.741	2.097	1.07-4.05	0.027
	Age	0.892	2.441	1.07-5.58	0.034
	NIHSS	1.224	3.401	1.77-6.55	0.000
	Smoking	-0.81	0.445	0.21-0.94	0.032
